# Introduction to the standard reference data of electron energy loss spectra and their database: eel.geri.re.kr

**DOI:** 10.1186/s42649-019-0015-3

**Published:** 2019-12-31

**Authors:** Jeong Eun Chae, Ji-Soo Kim, Sang-Yeol Nam, Min Su Kim, Jucheol Park

**Affiliations:** Test Analysis Research Center, Innovative Technology Research Division, Gumi Electronics & Information Technology Research Institute, Gumi, 39171 South Korea

**Keywords:** Electron energy loss spectroscopy, Electron energy loss data center, Standard reference, Transmission electron microscopy

## Abstract

Electron energy loss spectroscopy (EELS) is an analytical technique that can provide the structural, physical and chemical information of materials. The EELS spectra can be obtained by combining with TEM at sub-nanometer spatial resolution. However, EELS spectral information can’t be obtained easily because in order to interpret EELS spectra, we need to refer to and/or compare many reference data with each other. And in addition to that, we should consider the different experimental variables used to produce each data. Therefore, reliable and easily interpretable EELS standard reference data are needed.

Our Electron Energy Loss Data Center (EELDC) has been designated as National Standard Electron Energy Loss Data Center No. 34 to develop EELS standard reference (SR) data and to play a role in dissemination and diffusion of the SR data to users. EELDC has developed and collected EEL SR data for the materials required by major industries and has a total of 82 EEL SR data. Also, we have created an online platform that provides a one-stop-place to help users interpret quickly EELS spectra and get various spectral information. In this paper, we introduce EEL SR data, the homepage of EELDC and how to use them.

## Introduction

Electron energy loss analysis performed on transmission electron microscopy can provide the chemical and physical information of materials and is the only means to measure the electronic structure that can determine the properties of the materials at specific area with atomic-scale spatial resolution. And EELS analysis can identify almost all elements from hydrogen (H) to uranium (U) on the periodic table of the elements and also can measure the atomic numbers and elemental compositions of the compound materials. Therefore, the need of EELS analysis has increased because nanoscale analysis of key materials becomes increasingly important as the high-tech industry develops (Egerton 2009; Zhu et al. 2013).

The shape of EELS high-loss spectra is related with density of states, more specifically the unoccupied states in the conduction bands of the atoms that make up materials. By interpreting their shape, we can obtain chemical and physical information such as chemical bonding states and coordination number of atoms. So we can distinguish various materials composed of the same elements by using EELS analysis (Verbeeck and Aart 2004; Tan et al. 2012).

However, the analytical interpretation of EELS spectra is slightly complicated. In the case of low-loss EELS spectrum, its shape reflects the effects of several factors including volume plasmon, surface plasmon, inter-band transition and semi-core state transition. In the case of high-loss spectrum, the overlap of ionization edges, chemical shift, change of white-line L_3_/L_2_ ratio and near-edge fine structure can make it difficult to interpret the EELS spectrum. In order to accurately interpret EELS spectra, we should refer to as much reference data as possible through experimentation, theory calculation and literature survey, with prior knowledge of materials themselves. The most common way to interpret EELS spectrum is to compare it with already verified experimental data (Riedl et al. 2006; Lajaunie et al. 2015; Krivanek et al. 2019). However, it should be very careful because the reference data also could be obtained under different experimental conditions.

Up to now, the reference data commonly used for EELS analysis has been the data of the EELS Atlas book published in 1983 by the equipment manufacturer. However, some data from EELS Atlas have turned out to be relatively poor in terms of quality and accuracy with compared to data recently obtained with high-performance equipment showing excellent energy resolution and signal intensity. And there are also database (DB) systems of EELS reference data built in the U.S. (WEELS) and Europe (EELS DB). However, they have still limited reference data for application to high-tech industry and cutting-edge research. Therefore, the development of many reliable EELS data have been increasingly needed (P Ewels et al. 2016).

In this paper, we will introduce the some EEL standard reference (SR) data and our database system of 82 EEL SR data. How to use of EEL SR data of our database will be mentioned. More detailed explanation for the production of standard reference data will be published in another paper.

## Materials and methods

### Production of EELS reference data

EELS analysis was performed using a Gatan Quantum GIF965 dual EELS spectrometer attached to the transmission electron microscope (JEOL ARM-200CF) equipped with a cold field-emission electron gun and probe spherical-aberration corrector.

Reference materials used to maintain a traceability of specimen was NiO which has been traditionally used by EELS manufacturer. The samples had been kept uncontaminated, and carbon contamination was removed before and/or during the measurement. The measurement mode is STEM mode or diffraction mode. After checking the degree of carbon contamination and oxidation, in dual EELS mode, acquisition of EELS data was repeated five times in different locations and their average values of on-set energy were used. Then, the data was evaluated in accordance with the SR data evaluation procedures established by our data center. Detailed procedures and explanations will be mentioned in another paper.

### Construction of EELS database

**Fig. 1:**
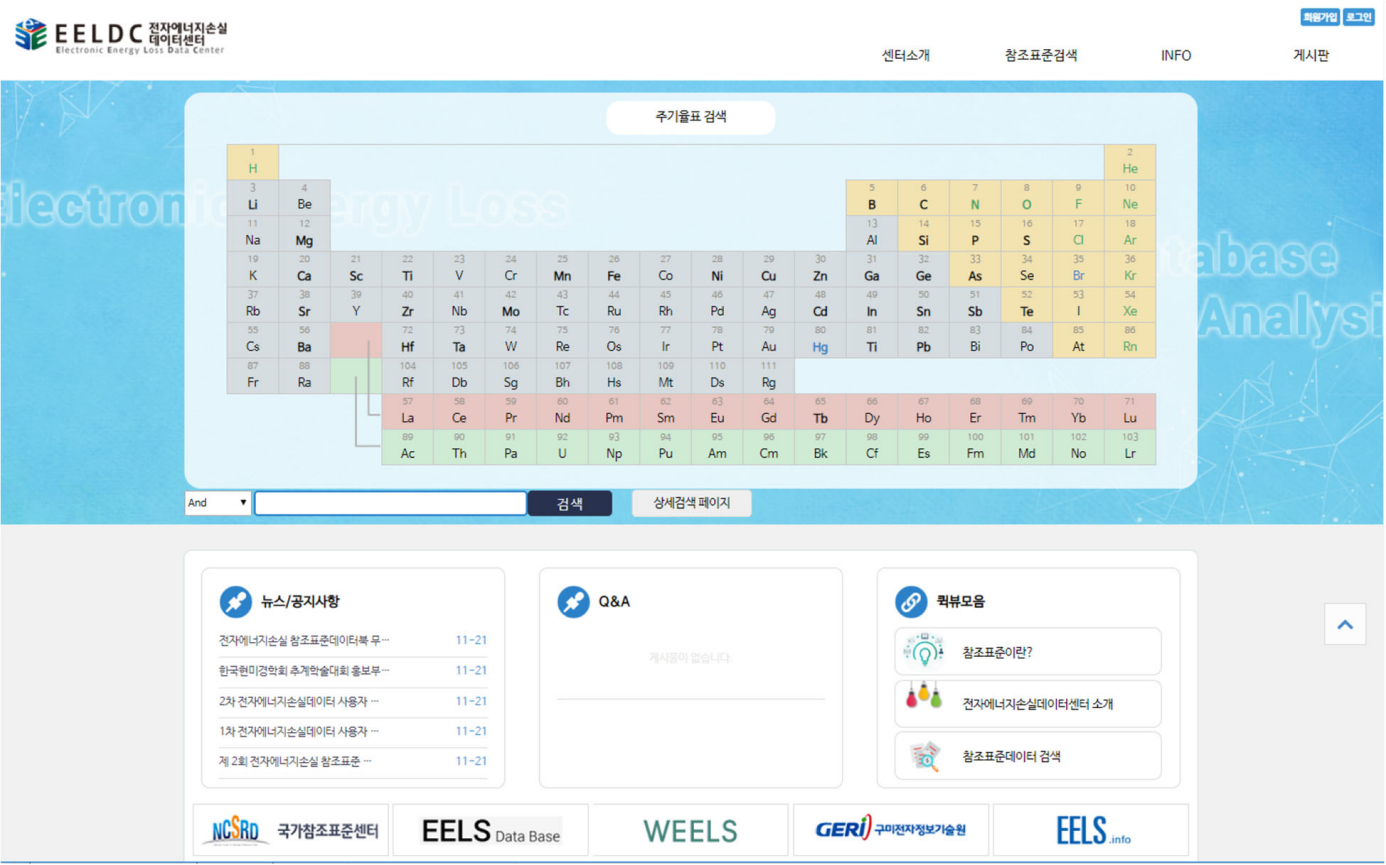
Main page of Electron Energy Loss Data Center (EELDC) website (http://eel.geri.re.kr)

We constructed the homepage (eel.geri.re.kr) of our EELS data center through HTML5-based reactive web (CSS) to improve database usability and has applied a user-friendly interface. To facilitate the search and utilization of EELS standard reference data, data search system was constructed based on the periodic table of elements displayed on the main screen of the website (Fig. 1). On the menu bar, the introduction of general standard reference data, EEL data center and EELS SR data, and another separate search function were prepared. In addition, maintenance functions such as bulletin manager and user manager were added to efficiently operate our website and improve it by reflecting feedbacks from users.

## Results and discussion

### Standard reference data of EELS

“Standard reference (SR) data” can be defined at the data that are certified through scientific analysis and evaluation of the accuracy and reliability of measurement data and information, and can be used continuously or repeatedly in all fields of national society. In short, the SR data should be recognized and shared as reliable reference data at the national level. In order to create a SR data, a quality system should be established to produce, collect and evaluate measurement data. In November 2016, our group was designated as the 34th National Reference Standard Electron Energy Loss Data Center (EELDC) to play a role in developing and disseminating EELS reference data by the Ministry of Trade, Industry and Energy of Korea.

All EELS SR data produced by EELS DC were acquired according to our standard procedures for EELS production certified by the National Reference Standards Center of Korea. All information related to measurement, such as samples, equipment specification and measurement conditions, was recorded in the meta-information table as shown in Table 1. Also, the SR EELS data were evaluated and certified based on the overall criteria of the data assessment provided by the National Reference Standards Center of Korea. Currently, EELDC has produced 82 SR EELS data which includes core materials needed in major industries such as semiconductors, displays and secondary batteries.

**Table 1:** Meta information of EEL SR data

**General**	
Element:	Ti
Formula:	TiO_2_
Name:	Titanium
Classes:	Element
**Specimen Information**	
Sample Production Method:	Standard Materials TED PELLA-18241
Specimen Preparation Method:	FIB
Specimen Type:	Bulk
Relative Thickness (t/λ):	0.73
**Data**	
Bandgap Energy (eV):	
Plasmon Energy (eV):	
**Equipment Information**	
Microscope Name / Model:	JEOL
Gun Type:	Cold FEG
Detector:	GIF965-ER
Acquisition Mode:	Diffraction mode
Convergence Semi-angle (mrad):	0.001
Collection Semi-angle (mrad):	10.4
Probe Size (nm):	
Beam Current (pA):	100
Incident Beam Energy (keV):	200
Best Energy Resolution (eV):	0.33
Incident Beam Energy (keV):	200
Energy Resolution (eV):	0.33
Vacuum status	1.10E-05
**Spectrum Information**	
Dispersion (eV/pixel)	0.05
Data Range (eV)	446–548
ZLP Resolution (eV):	0.65
High-Loss Exposure Time (sec):	2.0
Number of Readouts (times):	100
Edge Energy (eV):	457.4
Extended Uncertainty (eV):	0.6
**Spectrum Information**	
Dispersion (eV/pixel):	0.1
Data Range (eV):	436–641
ZLP Resolution (eV):	0.8
High-Loss Exposure Time (sec):	1.0
Number of Readouts (times):	100
Edge Energy (eV):	457.4
Extended Uncertainty (eV):	0.6
**Spectrum Information**	
Dispersion (eV/pixel):	0.25
Data Range (eV)	406–918
ZLP Resolution (eV):	1.5
High-Loss Exposure Time (sec):	0.5
Number of Readouts (times):	100
Edge Energy (eV):	457.4
Extended Uncertainty (eV):	1.0
**Data Correction**	
Dark Current Correction:	
Gain Variation Spectrum:	
Calibration:	
Deconvolution Methods:	
**Citation**	
Author Name(s):	
Journal:	
url site:	
**Notes**	
Carbon contamination: none	

Each EELS SR data consist of several spectra with different dispersions and meta-information which includes material name, element name, sample information, equipment information, measurement conditions and uncertainty values. The uncertainty values are provided to ensure measurement traceability and reliability of our measurements.

### Comparison between EELS atlas and SR EELS data

**Fig. 2:**
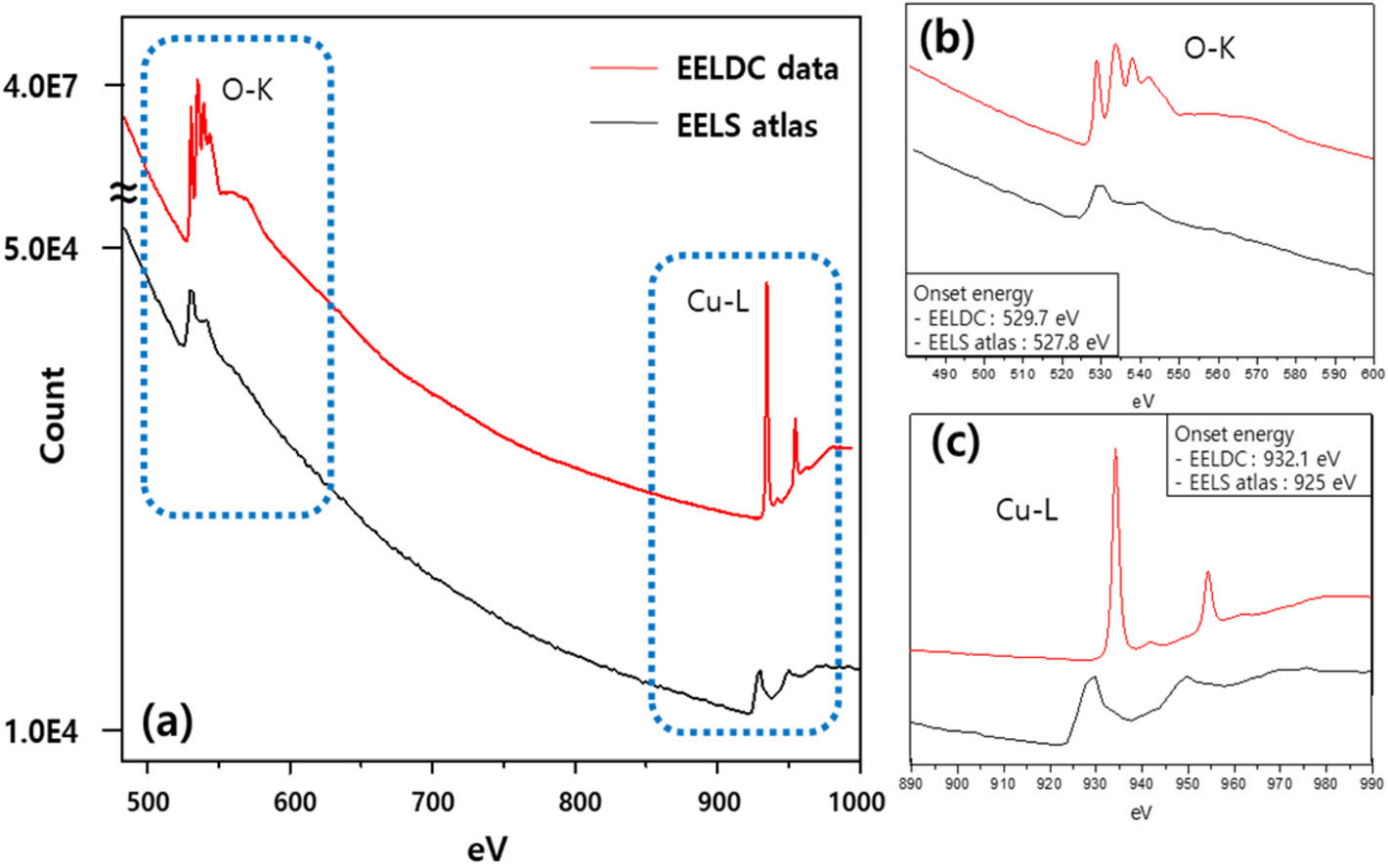
Comparison of (**a**) existing reference data (EELS Atlas) and the recent standard reference data (EELDC) of CuO. The magnified areas of O-K and Cu-L are shown in (**b**) and (**c**), respectively

Previous EELS reference data have showed some reliability issue due to poorer equipment performance and the lack of data for new materials compared to the most recent EELS reference data. As an example, the EELS spectra of CuO in the existing reference data (EELS Atlas) and in the recent SR data (EELDC) was compared in Fig. 2. They show spectral shape difference. It is possible because they are obtained by different acquisition condition such as energy range and beam intensity. However, onset energy position of O K-edge and Cu L-edge and details of their peak shape show inconceivable differences shown in Fig. 2b and c. EELS SR data show very sharp peak shape and exact onset position of O K-edge and Cu L-edge.

### How to use the data search system

We constructed an online database system of the 82 EELS SR data for immediate and convenient use. By using our database through web server, the interpretation of EELS spectra can be easily performed without the individual hassle of searching for structural information, characteristics or phase identification. Also, how to use EELS SR data and website homepage is designed to be relatively easy and convenient. Users who access the homepage are guided into four main categories, which are the introduction of our data center, SR data search, SR information and bulletin board at the top of the homepage. Through simple membership, users can get various useful information from each menu.

**Fig. 3:**
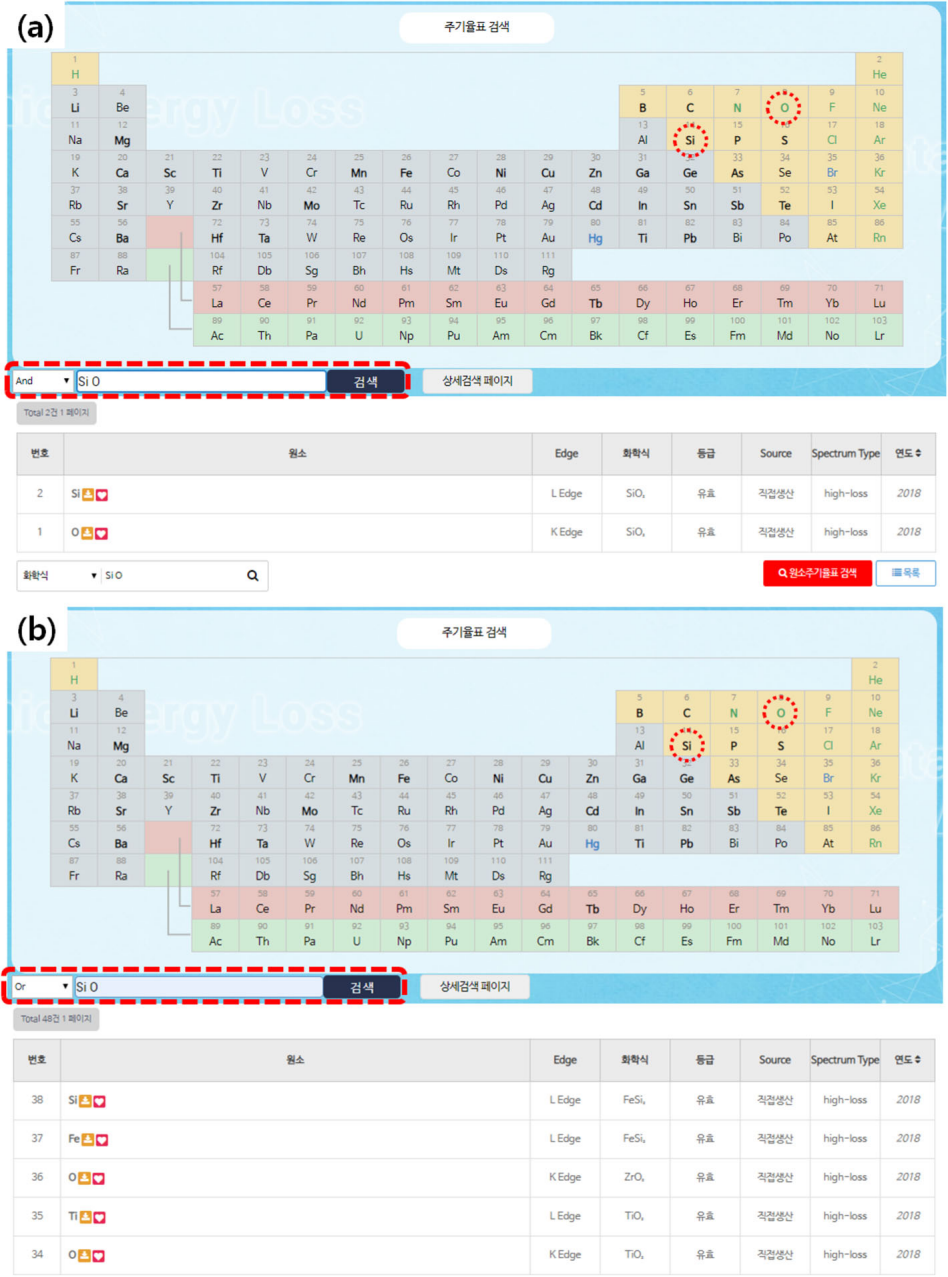
Data search methods and their retrieved results. **a** Searching for Si and O by selecting ‘and’ results in the materials containing both Si and O. **b** Searching for Si and O by selecting ‘or’ results in the materials containing Si or O

Data searching method using the periodic table of elements was adopted for intuitive and efficient use. The instruction manual of the periodic table search system is shown in Fig. 3. How to use is as follows: select the desired element in the periodic table and click ‘Search’. If the compound you are looking for consists of two or more elements, you can click ‘and’ and select another element of the compound. On the other hand, if you search by selecting ‘or’, all materials including the elements that you selected will be retrieved. Another data search function is shown in the menu of reference standard search.

**Fig. 4:**
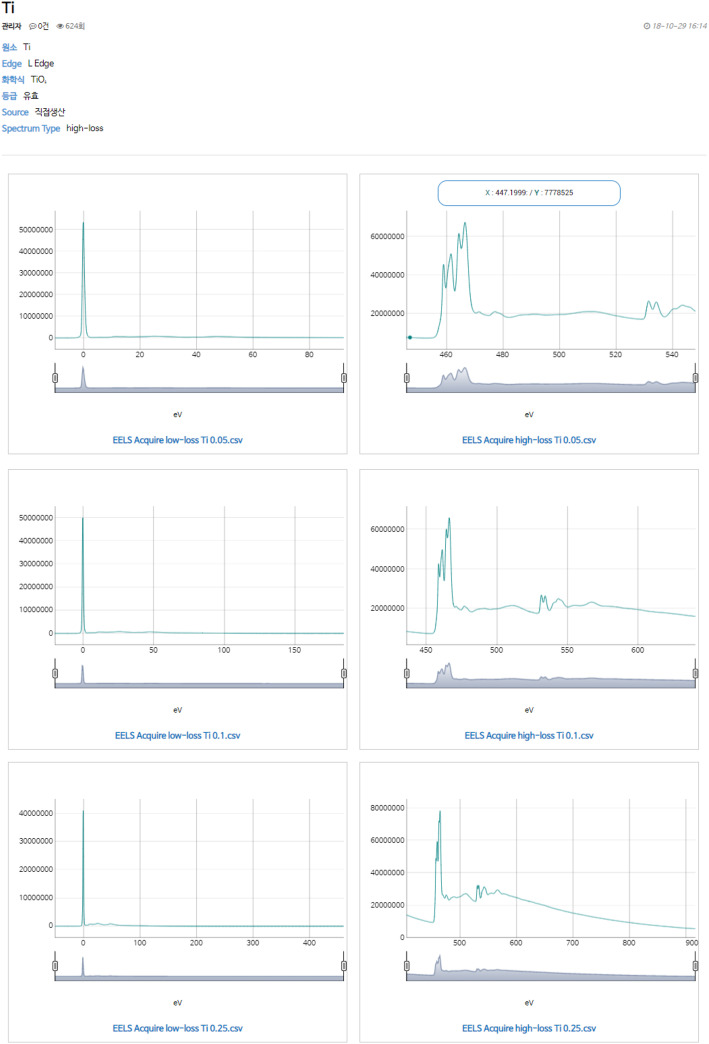
EEL standard reference spectra of Ti-L edge of TiO_2_ with the 0.05, 0.1 and 0.25 eV dispersion. CSV files can be downloaded by clicking the blue name below each spectrum

The retrieved list is represented by edge type, chemical formula, data class, production method and spectrum type. A searched data consist of various spectra with different dispersion (at least 2 to 3) and different energy range (low and high region). The spectra can be magnified by moving x-axis slide bar below the spectrum and the detailed shape of them can be observed. As x-axis range is freely selected, the y-axis is automatically adjusted according to the maximum intensity at each range. CSV files can be downloaded by clicking the blue name below each spectrum (Fig. 4). For users to process the spectra directly, DM files as raw data will also be provided.

Also, each spectrum data has its own meta-information consisting of general information, specimen information, equipment information and spectrum information (Table 1). In the spectrum information, specifically onset energy and measurement uncertainty values for data reliability and accuracy are provided.

### Using the homepage

Our homepage was organized according to itemized functions, such as the introduction of the data center and standard references, bulletin boards and the search system of EEL SR data as shown in Fig. 2. The homepage also provides a calendar (displaying the relevant event schedule), links to other EELS reference database homepage and user information management. The Electron Energy Loss Data Center has hold user training courses, forums, workshops and seminars to introduce various programs to promote and utilize EEL SR data. All schedules and results to the events are posted on the website, and users who want to participate in them can apply through our website. EELDC also has published annual EEL SR data books and has distributed them at the events hosted by the data center. If you want to get the data books, you can apply for it on the homepage or by e-mail. Related details will be posted on the homepage notice. The homepage of the Electron Energy Loss Data Center will be continually reorganized as required by users and ask their requirements and questions through bulletin boards or e-mail.

## Conclusion

We introduced the electron energy loss standard reference and the website of electron energy loss data center (eel.geri.re.kr). The electron energy loss standard reference (EEL SR) database is provided on the website of the electron energy loss data center and is easily accessible to users through the search system using the periodic table of elements. Eighty-two EELS SR spectra with various dispersions and detailed meta-information are provided to make it much easier to effectively utilize the data. The website was designed to be simple to zoom in and out the spectra online and check spectrum shape and energy values. The EELS SR data with detailed meta- information can be downloaded as CSV or DM files for users to conveniently process and analyze them.

## Data Availability

The datasets used and/or analyzed during the current study are available from the corresponding author on reasonable request.

## Abbreviations

EELDC: Electron Energy Loss Data Center
EELS: Electron energy loss spectroscopy
SR: Standard reference
STEM: Scanning transmission electron microscope
